# A method for determining total nitrogen in Kjeldahl digestion solution using a centrifugal analyser

**DOI:** 10.1155/S1463924687000166

**Published:** 1987

**Authors:** J. W. Geiger, N. M. Davis, W. S. Blakemore, C. L. Long

**Affiliations:** Research Division Baptist Medical Center Princeton 701 Princeton Avenue Birmingham Alabama 35211 USA


Journal of Automatic Chemistry/*Journal of Clinical Laboratory Automation*, Vol. 9, No. 2 (April–June 1987), pp. 72–76

### A method for determining total nitrogen in Kjeldahl digestion solution using a centrifugal analyser

**J. W. Geiger, N. M. Davis, W. S. Blakemore and C. L. Long**
*Research Division, Baptist Medical Center Princeton, 701 Princeton Avenue, Birmingham, Alabama 35211, USA*

### Introduction

Protein requirements are classically determined by assuming that nitrogen losses from the body represent individual requirements. These losses may be used to evaluate the protein needs of an individual, either healthy, malnourished, or hypercatabolic. When a large and representative population is evaluated with respect to total nitrogen output, an approximation of recommended daily allowances can be made. For these recommendations, the average nitrogen loss is tempered to cover all variants in a given population using two standard deviations and the mixed protein intake. Protein is assumed to have a biological value of 70.

Currently, the protein status of a hospitalized patient is evaluated by determining the urea nitrogen content of a 24-hour urine collection. The urea nitrogen value is extrapolated to total nitrogen using a correction factor as described by Blackburn [1]. The nitrogen content of the stool is not determined when this procedure is followed, but this method has been quite useful in approximating protein needs as urinary urea nitrogen is routinely assayed in most hospital laboratories. The evaluation of protein status would be more accurate if the total nitrogen of both urine and stool were used. The original Kjeldahl method was too time-consuming and was suitable only in a research setting.

A method for the rapid determination of total nitrogen in urine and stools would be useful in evaluating the protein needs of the hospitalized patient. A nitrogen analyser is available that combusts small samples with subsequent automatic gas analysis using chemoluminescence. Its use has been reported to be suitable for analysis of total nitrogen in urine; however, there have been some technical problems in solid-sample analysis as well as with interfering components in certain urines. This method is rapid, but would require an expensive instrument dedicated only to nitrogen analysis.

If a colorimetric ammonia methodology could be adapted to a multi-use analyser capable of rapid analysis, then it would be possible to quantitate total nitrogen in urine and stools in the hospital setting, probably utilizing equipment already available. We decided to adapt the colorimetric ammonia methodology of Russell [2] to the Encore Centrifugal Analyzer (Baker Instruments Corporation, Allentown, Pennsylvania, USA) since the analyser is capable of analysing small numbers of samples rapidly. In addition, the centrifugal analyser requires micro volumes of reagents.

The traditional Kjeldahl method of digesting organic nitrogen compounds is a heated wet digestion process during which the compounds are converted to ammonium sulphate. The digested sample is made to volume with water and assayed for ammonia.

There are two basic methods for determining the ammonia concentration in the digested and diluted sample. One is steam distillation into a receiver flask containing a quantitative amount of alkali with subsequent back titration with standardized acid. The concentration of ammonia is calculated using the standard acid-base relationships of milliliters, normality, and milliequivalents. The ammonia formed in the digestion process may also be determined colorimetrically. The reaction of ammonia with alkaline phenol and sodium hypochlorite forms a dark blue colour that is proportional to the concentration of ammonia present. This colour reaction was adapted to flow chemistry analysers, such as Technicon (Technicon Instruments Corporation, Tarrytown, New York, USA). The method required large reagent volumes and was useful for large numbers of samples. It was not practical for single sample analysis.

### Methods

Adapting methodology to a centrifugal analyser is more than scaling down macro volumes to micro volumes. The mixing of reagents with a sample in the cuvette of the rotor is very rapid. Because the analytical volume is very small, temperature equilibration of the reaction mixture is achieved very quickly. These factors often reduce the time it takes to reach an end-point.

There are 30 cuvettes on the analytical rotor. One cuvette contains the thermister probe and is used to monitor the temperature of the rotor; water is automatically pipetted into this cuvette by the automatic pipettor. A second cuvette is utilized as a reagent monitor. Reagents and water are pipetted into this cuvette whose purpose is to establish a baseline reading for the reagents. The milliabsorbance value of each cuvette is read by the microprocessor each time a data point is taken. The milliabsorbance value of the reagent cuvette is subtracted from the milliabsorbance value of all other cuvettes (except the thermister cuvette) every time a data point is taken. Data points are taken every 2 s for every cuvette throughout the run.

The analyser has a research mode that is useful for developing methods. In the research mode, there is no manipulation of data points, no curve fitting, no data reduction, and no blank subtraction (unless desired). The developing reaction can be observed on the video monitor. After the reaction is complete, the curve can be evaluated on the video monitor by modifying either the time and/or the milliabsorbance scale.

The analyser determines concentration by using the delta change in absorbance over a period of time between two points on a curve. Determining where on the curve these two points should be taken can be estimated using the research mode of the analyser because the reaction may be observed. The rate of this reaction is rapid during the first 30 s and then it slowly plateaus.

All reagents used were of an analytical grade. The digestion mixture, adapted from the Technicon methodology, was as follows:

| | |
|---|---|
| Selenium dioxide | 3 g |
| Sulphuric acid, Conc. | 900 ml |
| Perchloric acid, 70% | 20 ml |

Make to 1000 ml volume with distilled water.

The selenium dioxide was dissolved in about 50 ml distilled water and the perchloric acid added slowly. Then, the mixture was placed in an ice-bath over a magnetic stirrer and the sulphuric acid was added slowly. Next, the digestion mixture was made to volume with distilled water, and stored in a brown glass bottle fitted with a repeating pipette.

The wet digestion of standards, controls, and samples was carried out in 100 ml volumetric glass tubes in a Digestion Block (Tecator, Inc., Herndon, Virginia, USA). One half ml or 1·0 ml of standards, controls, or urine was placed in the digestion tube. Two ml of digestion fluid was added. One glass bead was added to prevent bumping and the tube contents were digested for 1 h at 425 °C. After digestion, the tubes were cooled to room temperature and made to volume with distilled water.

Reagents for the colorimetric reaction were modified from Russel [2]. They were prepared as follows:

(1) Sodium hypochlorite (Clorox) was diluted one-to-two with water and stored in the refrigerator.

(2) Alkaline phenol: 16 ml 20% sodium hydroxide in water was added slowly to 13 ml liquid phenol. The mixture was cooled and stored in a brown glass bottle.

(3) Acidified water: 1·0 ml distilled water and 2·0 ml digestion fluid were digested for 1 h then diluted to 100 ml with water.

A standard curve ranging from 1 to 5 mg % of nitrogen was prepared from a stock solution of 100 mg % ammonium sulphate by dissolving 0·47192 g ammonium sulphate in 100 ml distilled water. Working standards were prepared by pipetting appropriate volumes of the stock solution to 100 ml digestion tubes. These tubes were dried overnight in a gravity oven at 70 °C. When dry, 1·0 ml water and 2·0 ml digestion fluid were added to each tube to assure that standards would be treated as samples. The tubes were digested as described above. Quality controls were prepared and run through the entire process, including digestion. These controls were:

(*a*) 300 mg % ammonium sulphate prepared by dissolving 1·41576 g ammonium sulphate in 100 ml distilled water.

(*b*) 300 mg % urea prepared by dissolving 0·64299 g urea in 100 ml distilled water.

Digestion of 1·0 ml of either the ammonium sulphate solution or the urea solution made to 100 ml volume should read 3 mg % against the standard curve.

A urine sample for quality control was aliquoted into 2 ml portions and frozen. One half ml of this urine is digested and assayed during each run, and a quality-control record is maintained for each of the above quality-control solutions.

Digestion of 0·5 ml of a urine sample is usually appropriate. Occasionally a sample must be rediluted or a larger amount digested. Assuming a 24 h urine volume of 2000 ml with a total nitrogen content of 10 g, digestion of 0·5 ml when made to a volume of 100 ml would be 2·5 mg %. Twenty-four hour urine volumes and known nitrogen intake may be used to calculate appropriate sample size for digestion. Parameters used in the analytical mode of the system are shown in table 1.

### Results and discussion

The reaction of ammonium sulphate with alkaline phenol and the subsequent colour development with sodium hypochlorite (Clorox) is a very rapid reaction. The curve begins to plateau after 60 s and continues to increase slowly in milliabsorbance values. It appears that the reaction is still changing even after 16 min; however, it is almost complete at this point (slope = 0·27). This is shown in figure 1.

Russel [2] and Borsook [3] indicate that it takes from 1 and 2 h for the reaction to be complete at 37 °C. Shorter time intervals were not reported. We have not evaluated time and temperature beyond 999 s and 37 °C, 999 s being the time limit of the Encore. It was necessary to define a portion of the curve where the reaction was linear at the same time for each of the standards. The curves in figure 1 show that the point where the reaction is about 70% complete occurs at 30 s, and 95% complete at 8 min. With this system, we had the option determining the change in optical density between 2 s and 30 s where the reaction was 70% complete or continue to 8 min where the reaction was 95% complete. 480 s was selected as the beginning point for calculations based on sensitivity and reproducibility of repetitive assays.

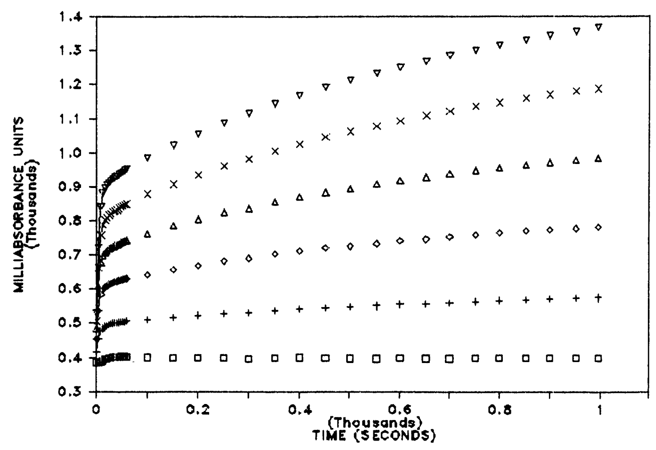

*Figure 1. Standard nitrogen curve showing reaction from zero to 16 min for 1, 2, 3, 4, 5 mg % ammonium sulphate and reagents. Where + = 1 mg %; ◇ = 2 mg %; △ = 3 mg %; x = 4 mg %; ▽ = 5 mg %; and □ = reagents.*

A graph of the 3 mg % standard at six different wavelengths is shown in figure 2. It was decided to use 620 nanometers as an appropriate wavelength as this available wavelength provided maximum delta absorbances when the cuvettes were read against water as previously reported by Van Slyke [4]. This centrifugal analyser has three temperature options: 25 °C, 30 °C and 37 °C. Although the Encore can monitor these fast reaction rates (less than 30 s) temperatures below 37 °C were evaluated to determine if the reaction might provide end-points at about 1 to 2 min. These trials systematically produced more variation in absorbance changes compared to using 37 °C and reading the end-point at 8 min. Russel [2] evaluated temperature and pH, and concluded that pH was the major factor in colour development. Borsook [3] also used 37 °C in his micro ammonia method.

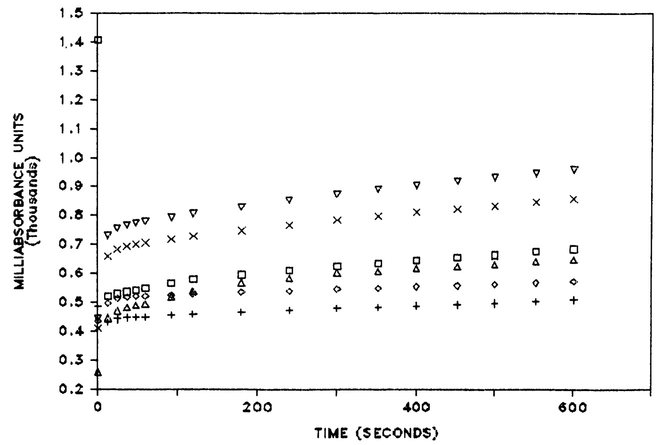

*Figure 2. 3 mg % ammonium sulphate curve shown at various wavelengths. Where □ = 420 nm; + = 500 nm; ◇ = 520 nm; △ = 580 nm; x = 600 nm; and ▽ = 620 nm.*

Initially, the macro reaction was scaled down to microliter volumes for the Encore. After evaluating these runs, the concentrations of the reagents and the sample size were varied. The effect of variation of the concentration of both the Clorox and the alkaline phenol is shown graphically in figure 3. These variations were arbitrary since all that was effectively being changed was the pH. The criteria for evaluating these reagent changes was the colour yield as measured in milliabsorbance units at 620 nm. In addition, the precision of known samples read against the standard curve were evaluated. The final sample and reagent parameters were as follows:

| | |
|---|---|
| Sample | 50 μ |
| Sodium hypochlorite (Clorox) | 160 μ |
| Alkaline phenol | 80 μ |
| Acidified water | 30 μ |

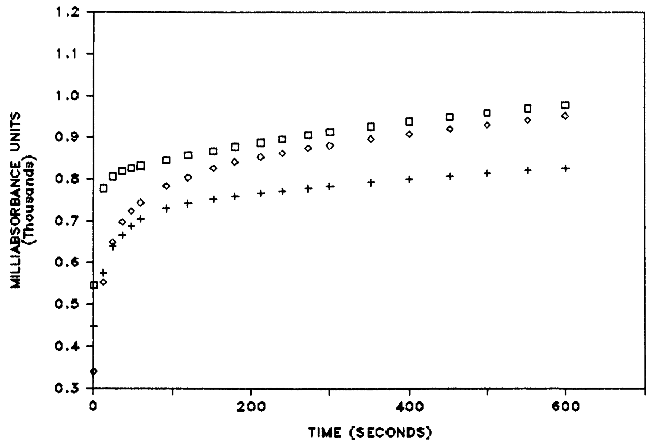

*Figure 3. Effect of varying concentrations of alkaline phenol and sodium hydroxide on the 3 mg % nitrogen curve. Where □ = 16 ml 20% sodium hydroxide + 13 ml phenol; + = 16 ml 10% sodium hydroxide + 13 ml phenol; ◇ = 16 ml 20% sodium hydroxide + 6·5 ml phenol + 6·5 ml water.*

*Table 1. Analytical parameters for the centrifugal analyser.*

| | |
|---|---|
| Test Name — Nitrogen | Light level — Normal (A) |
| Temperature = 37 °C | Optical signal — Absorbance (A) |
| | Test Type — End |
| Point (C) standards | 1·00 mg/dl |
| Test units — mg/dl | 2·00 mg/dl |
| | 3·00 mg/dl |
| Reaction direction — | |
| Increasing (A) | 4·00 mg/dl |
| Low pass filter — Low (A) | 5·00 mg/dl |
| | Y-transforms — None (A) |
| Wavelengths — | Curve fit — Polynomial of |
| Analytical — 620 (1) | order 1 (C) |
| Blank 1 — 620 (1) | X — Transforms — None (A) |
| Blank scale factor | Note — ( ) Designates letter to |
| Blank A - 1·00 | choose for appropriate |
| | parameter |
| Blank 2 - 0 | |
| Blank 3 - 0 | |
| Mode — Stored (B) | |
| | Analysis times |
| TI — 460 | Linearity - 1·0 |
| TW — 50 | Abnormal absorbance limit — 1·0 |
| | TF — 660 |
| Concentration factor — 0 | |
| Mix time — 1·6 | Absorbance threshold - 0 |
| Linearity — 1·0 | |

It was necessary to use acidified water as a diluent in the pipetting of reagents and samples on the pipettor. The pipetting sequence is such that the alkaline phenol is deposited in the outer well of the transfer disk before the sample. There is a carry-over of alkali on the sample tip which is rinsed into the diluent cup of the boat. This contaminated the diluent and was sufficient to alter the pH of the reaction system. Using acidified water resolved the problem.

*Table 2. Coefficients of variation for ammonium sulphate standards.*

| Milligram % | Mean | SD | CV |
|---|---|---|---|
| 1 | 1·00 | 0·03 | 3·90 |
| | 1·00 | 0·03 | 3·10 |
| | 1·02 | 0·04 | 3·91 |
| Mean | 1·01 | 0.03 | 3·63 |
| 2 | 2·06 | 0·05 | 2·84 |
| | 2·08 | 0·04 | 1·99 |
| | 2·02 | 0·03 | 1·57 |
| Mean | 2·05 | 0·04 | 2·13 |
| 3 | 2·99 | 0·09 | 3·29 |
| | 3·03 | 0·06 | 2·08 |
| | 3·05 | 0·05 | 1·69 |
| | 3·07 | 0·05 | 1·88 |
| | 3·05 | 0·06 | 2·16 |
| | 2·99 | 0·05 | 1·86 |
| Mean | 3·03 | 0·06 | 2·16 |
| 4 | 4·08 | 0·10 | 2·60 |
| | 3·99 | 0·07 | 1·74 |
| | 4·08 | 0·07 | 1·76 |
| | 4·08 | 0·10 | 2·57 |
| Mean | 4·06 | 0·08 | 2·16 |
| 5 | 5·01 | 0·06 | 1·29 |
| | 5·09 | 0·07 | 1·43 |
| Mean | 5·02 | 0·06 | 1·36 |

* Each entry represents 28 determinations.

*Table 3. Coefficient of variation in total nitrogen determinations in a urine quality-control sample.*

| Number of samples | Mean milligram % | SD | CV |
|---|---|---|---|
| 28 | 2·79 | 0·05 | 1·96 |
| 28 | 2·91 | 0·08 | 2·74 |
| 28 | 2·79 | 0·05 | 1·99 |

Having established the appropriate conditions using the research mode, it was then necessary to establish parameters for the analytical mode that would be used for routine sample analysis. Based on the rate of reaction (figure 1), the analyser was programmed to begin taking data points for analysis at 460 s and to stop taking data points at 660 s.

Reading the cuvettes against water uses the 'hold blank' feature of the analyser since on immediate mixing of the reagents and sample, the first reading available at a 4 s was well into the reaction. Larger optical density changes were required for a given ammonia concentration and therefore this 'hold blank' modality was selected. Water is loaded by the pipettor into the transfer disk and processed as in a regular run. The milliabsorbance values from this run are stored in the microprocessor. The analyser is programmed to read future runs against these water blanks.

In developing a method, it is necessary to know both the sensitivity and accuracy of the method. Coefficients of variation were determined for each of the five standards by running a full transfer disk (28 replicates) for each standard. Each of the mean values in the table 2 represents 28 determinations at that particular level of standard. Coefficients of variation were also run for the urine quality control as seen in table 3. Again, each mean represents 28 determinations.

For both the standards and the urine control, the coefficient of variation was within 4%. An analytical method should be able to 'recover' a known amount of substrate added to a sample. This recovery should be within the analytical variation of the method and should be reproducible. Recovery data are shown in table 4.

Once the sample is digested and made to volume, the time required for the ammonia determination is minimal. Although this method requires more time then a clinical urea nitrogen analysis or a dedicated nitrogen analyser, the data developed by this technique is more accurate since it uses the total nitrogen of the urine and stool samples for the determination of the protein status of

*Table 4. Recovery of a known amount of nitrogen added to a urine sample.*

| Solution | Ml digested | Calculated | Found | % Recovered |
|---|---|---|---|---|
| Urine No. 1 | 1·0 | — | 2·27 | — |
| Urine 1:2 with Water | 1·0 | 1·14 | 1·11 | 97·4 |
| Urine 1:2 with 600 mg % $(NH_4)_2SO_4$ | 1·0 | 4·14 | 4·08 | 98·6 |
| Urine No. 2 | 0·5 | — | 2·95 | — |
| Urine 1:2 with Water | 0·5 | 1·48 | 1·44 | 97·3 |
| Urine 1:2 with 1200 mg % $(NH_4)_2SO_4$ | 0·5 | 4·48 | 4·51 | 100·6 |

patients. After 1 h of digestion, which is possible using the current digestors, the digested samples can be analysed concurrently with other chemistries using the multi-chemistry feature of the Encore. Self-contained digesters are available that may be used on the bench-top without venting to the outside atmosphere. They usually require a water aspirator and standard electrical power.

### Conclusion

Data for adapting a colorimetric ammonia methodology to a centrifugal analyser adaptable to a clinical setting have been presented. These data include guidelines for utilizing the research mode of the Encore centrifugal analyser to initialize parameters. In addition, we have presented precision and repetitive run data obtained after establishing analytical parameters for the analytical mode of the analyser.

### References

1. Blackburn, G. L., *Journal of Parenteral and Enteral Nutrition*, **3** (1977), 11.
2. Russel, J. A., *Journal of Biological Chemistry*, **156** (1944), 457
3. Borsook, H., *Journal of Biological Chemistry*, **110** (1935), 481
4. Van Slyke, D. D., *Journal of Biological Chemistry*, **102** (1933), 499

**CLUB DATA-BASE AVAILABLE**

One of the main objectives of the Chemical Sensors Club is to provide means by which members can exchange information. So far this has been achieved by formal meetings, newsletters, printed material and workshops, but these are being supplemented by a new data-base which is now available to Club members.

Membership of the Club is open to any company with an interest in chemical sensors, in the broadest sense of the term. Current interests are very diverse and cover such areas as:

- Research and development of chemical sensors.
- The production and sale of sensors.
- The use and application of sensors.
- Sensor consultancy.
- Providing finance for sensor research.

The Club believes, therefore, it is important that effective ways of liaising between members should be made available.

The new data-base provides another of these and contains information on the interests and activities of members of the Club as described by the individual members themselves (based on information supplied via the questionnaire given to members on joining). The recorded description of a typical member covers the following areas:

- Category of the member's organization. This has been classified into about 10 groups such as 'consultant', 'academic', 'supplier'; a member may well of course come into more than one category.
- Field of interest. This covers the area within which the work or activity is applied, for instance, 'medical', 'process industries'.
- Technology employed. This describes the techniques or types of measurement being used by a member or in which there is an interest, for instance, 'acoustics', 'electrochemical', 'biosensing'. At present there are about 36 categories of 'technology'
- Areas of research and facilities. This part of the data-base allows for a rather more extended textual description of the areas of chemical sensors in which a member is researching or is proposing to research. It also gives an account of the test and other facilities available.

Club members can access the data-base from their own terminal or PC.

The data-base itself is menu-driven, making it simple to handle, the objective being to enable members to identify, rapidly and easily, those other members with interests and activities similar, supportive or complimentary to their own. The data-base is continually being updated and improved. In this way contacts can be established which are of mutual benefit and speed up the process of technology transfer in the field of chemical sensors, in line with the philosophy of the club.

*Details of access to an operation of the data-base will be included in the Club Members' Handbook: copies from Ric Treble, LGC, Cornwall House, Waterloo Road, London SE1 8XY.*

